# MicroRNA-1224 Inhibits Tumor Metastasis in Intestinal-Type Gastric Cancer by Directly Targeting FAK

**DOI:** 10.3389/fonc.2019.00222

**Published:** 2019-04-04

**Authors:** Jin Wang, Ti Wen, Zhi Li, Xiaofang Che, Libao Gong, Xianghong Yang, Jingdong Zhang, Huali Tang, Lingzi He, Xiujuan Qu, Yunpeng Liu

**Affiliations:** ^1^Department of Medical Oncology, The First Hospital of China Medical University, Shenyang, China; ^2^Key Laboratory of Anticancer Drugs and Biotherapy of Liaoning Province, The First Hospital of China Medical University, Shenyang, China; ^3^Department of Pathology, Shengjing Hospital of China Medical University, Shenyang, China; ^4^Department of Medical Oncology, Cancer Hospital of China Medical University, Liaoning Cancer Hospital and Institute, Shenyang, China; ^5^Department of Medical Oncology, The Central Hospital of Zhuanghe, Zhuanghe, China

**Keywords:** intestinal-type gastric cancer, miR-1224, FAK, metastasis, epithelial-to-mesenchymal transition (EMT)

## Abstract

Intestinal-type gastric cancer (GC) of the Lauren classification system has specific epidemiological characteristics and carcinogenesis patterns. MicroRNAs (miRNAs) have prognostic significance, and some can be used as prognostic biomarkers in GC. In this study, we identified miR-1224 as a potential survival-related miRNA in intestinal-type GC patients by The Cancer Genome Atlas (TCGA) analysis. Using quantitative real-time PCR (qRT-PCR), we showed that the relative expression of miR-1224 was significantly decreased in intestinal-type GC tissues compared to matched adjacent normal mucosa tissues (*p* < 0.01). We found that high miR-1224 expression was associated with no lymph-node metastasis (*p* < 0.05) and good prognosis (*p* = 0.028) in 90 intestinal-type GC tissues. Transfection of intestinal-type GC cells with miR-1224 mimics showed that miR-1224 suppressed cell migration *in vitro* (wound healing assay and Transwell migration assay), whereas the transfection of cells with miR-1224 inhibitor promoted cell migration *in vitro*. miR-1224 also suppressed intestinal-type GC cell metastasis in a xenograft mouse model. Furthermore, bioinformatics, luciferase reporter, Western blotting, and immunohistochemistry (IHC) studies demonstrated that miR-1224 directly bound to the focal adhesion kinase (FAK) gene, and downregulated its expression, which decreased STAT3 and NF-κB signaling and subsequent the epithelial-to-mesenchymal transition (EMT). Repression of FAK is required for the miR-1224-mediated inhibition of cell migration in intestinal-type GC. The present study demonstrated that miR-1224 is downregulated in intestinal-type GC. miR-1224 inhibits the metastasis of intestinal-type GC by suppressing FAK-mediated activation of the STAT3 and NF-κB pathways, and subsequent EMT. miR-1224 could represent an important prognostic factor in intestinal-type GC.

## Introduction

Gastric cancer (GC) is one of the most common cancers worldwide with 70% of the total cases diagnosed in developing countries (1, 2). Although surgical resection is the primary curative treatment modality for GC, two-thirds of GC patients are diagnosed with metastatic disease (3, 4). Therefore, diagnostic and prognostic biomarkers of GC are greatly needed.

Although the Lauren classification system dates back to 1965, it still has important prognostic significance, and is widely accepted and employed by pathologists and physicians (5). The host epigenetic characteristics of the Lauren classification, such as genetic background and expression of microRNAs (miRNAs), is not fully clear (6–9). miRNAs are small non-coding RNAs that can tolerate ribonuclease degradation, so they can be completely preserved in formalin-fixed paraffin-embedded (FFPE) and plasma specimens. miRNAs are proper to be acted as prognostic biomarkers because of their molecular biological behavior characteristics (10, 11). Previous studies showed that miR-451, miR-10b, let-7a, miR-223, miR-21, and miR-338 are related to worse overall survival in GC (12–14). Other studies showed that miR-125a-5p, miR-34b, and miR-129-3p were biomarkers of good prognosis in GC (15, 16). The significance of miRNAs in the clinical characteristics of intestinal-type GC has only been investigated in the clinical characteristic part (14), however, there have been no studies that have explored the prognostic significance of miRNAs in this specific GC type.

In this study, we identified miR-1224 as an important prognostic biomarker for intestinal-type GC using The Cancer Genome Atlas (TCGA) analysis. Our results showed that miR-1224 suppressed tumor metastasis by directly targeting focal adhesion kinase (FAK) in intestinal-type GC and inhibiting the STAT3 and NF-κB signaling pathway, and subsequent epithelial-to-mesenchymal transition (EMT).

## Materials and Methods

### Bioinformatics

Analysis of potential survival related miRNAs in intestinal-type GC was performed using TCGA databases (https://www.cancer.gov/about-nci/organization/ccg/research/structural-genomics/tcga, up to Apr 7, 2016). Analysis of potential miR-mRNA interactions was performed using three common miRNA target prediction engines (TargetScan, PicTar, and RNA22). Involvement of target genes of KEGG pathway enrichment analysis was evaluated using DAVID online software (https://david.ncifcrf.gov/).

### Cell Cultures and Tissue Samples

The cells were all purchased from the Shanghai Chinese Academy of Sciences, and cultured in RPMI media 1640 (Gibco, MA, USA) supplemented with 10% fetal bovine serum (Thermo Scientific MA, USA) and 100 units/ml penicillin-streptomycin at 37°C containing 5% CO_2_.GC and paired adjacent non-cancerous gastric FFPE tissue (not <20 mm away from GC) specimens were obtained from Shengjing Hospital of China Medical University, Cancer Hospital of China Medical University and the Central Hospital of Zhuanghe City. All specimens were histopathologically confirmed with intestinal-type GC.

### RNA Isolation and Quantitative Real-Time PCR

Total RNA was extracted from culture cells using Trizol reagent (Invitrogen, Carlsbad, CA, USA) and from each FFPE GC tissues with a miRNeasy FFPE kit (Qiagen, USA) using a QiaCube automated platform (Qiagen), following the manufacturer's protocol. The extracted RNA was quantified by absorbance at 260 nm and the purity was evaluated by the absorbance ratio at 260/280 nm with a NanoDrop ND-100 spectrophotometer (NanoDrop Technologies, Rockland, DE, USA). The RNA was transcribed into cDNA using One Step PrimeScript ® miRNA cDNA Synthesis kit (Takara, Japan). Relative expression of miRNA was calculated via the 2^−ΔΔCT^ method after normalization with U6 small nuclear RNA.RT- PCR was carried out in the ABI PRISM 7500 system using the kit. SYBR® Premix Ex Taq™ II (Takara, Japan). The PCR conditions were 15 s at 95°C, followed by 45 cycles at 95°C for 15 s and 58°C for 34 s. The PCR primers used were as follows:

miR-1224: 5′-GTGAGGACTCGGGAGGTGGAAA- 3′;
U6 Forward: 5′-GCTTCGGCAGCACATATACTAAAAT-3′;
U6 Reverse: 5′-CGCTTCACGAATTTGCGTGTCAT-3′.

### Transient Transfection

The siRNA against FAK, miR-1224 mimics, miR-1224 inhibitor, and the corresponding NC were designed and synthesized from RiboBio (Guangzhou, China). The overexpression plasmids containing whole coding sequence of FAK and pcDNA 3.1 vector served as the NC were purchased from GeneChem (Shanghai, China), and we use pcDNA3.1-FAK to represent overexpressed FAK.

The sequences of siRNA were as follows:

siFAK-1:5′-CAGGUGAAGAGCGAUUAUATT-3′;
siFAK-2: 5′-CUCCAGUCUACAGAUUUGATT-3′;
NC siRNA: 5′-AATTCTCCGAACGTGTCACGT-3′.

SiFAK-1 was more effective than siFAK-2 (Figure S1), thus, all subsequent experiments were performed with siFAK-1. Target cells were transfected with siFAK, miR-1224 mimics, inhibitor, or the corresponding NC at a final concentration of 50 nM (mimics) or 100 nM (inhibitor) using Lipofectamine 2000 (Invitrogen, CA, USA) according to the manufacturer's protocol.

### Wound Healing Assay and Transwell Migration Assay

MKN-7 and MKN-28 cells were seeded in six-well plates overnight. Then, the cells were transfected with miR-1224 mimics or inhibitor, and the corresponding NC for 48 h. The cells grew to nearly 100% confluence in six-well plates. Then, the cell-free line was manually created by scratching the confluent cell monolayers using a 200-μl pipette tip. We randomly choose five scratched fields. The images were captured through bright-field microscope.

The Transwell migration assay was performed using Transwell chambers (8-μm pore size; Corning, USA). Briefly, 2 × 10^4^ cells were plated in serum-free medium onto the upper compartment of the chamber. Medium containing 5% fetal bovine serum was added to the lower compartment as a chemo-attractant. The plates were incubated for 24 h at 37°C. Then, the porous inserts were removed with a cotton swab and the cells that had migrated into the lower surface of the filters were fixed, stained with Trypan Blue and counted in 5 random fields under a microscope (magnification ×200). The experiments were all repeated three times.

### Western Blot Analysis

Western blot was conducted as previously described (17), using primary anti-bodies against the following proteins at the indicated dilutions: FAK, phospho-FAK Tyr 397, STAT3, phospho-STAT3, P65, phospho-P65, E-cadherin, vimentin (Cell Signaling Technology); and ZEB1, GAPDH (Santa Cruz). Secondary goat anti-rabbit and goat anti-mouse antibodies were purchased from Santa Cruz Biotechnology (Santa Cruz, CA, USA). The relevant reagents were shown as follows:Bay 11-7082 (NF-κB inhibitor) was obtained from Sigma. Stattic (STAT3 inhibitor) was obtained from Selleck. PF573228 (FAK inhibitor) was obtained from TOCRIS. The relative integrated density values (IDVs) were calculated based on GAPDH as an internal control.

### Luciferase Plasmid Construction and Luciferase Reporter Assay

Luciferase reporter gene assays were performed using the Dual-Luciferase Reporter Assay System (Promega, USA), according to the manufacturer's instructions. The 3′UTR containing the predicted miR-1224 binding sites wild-type (WT) and mutated (MUT) 3′UTR of the FAK gene (OriGene). The cells were co-transfected with miR-1224 mimics and the luciferase reporter vector. The luciferase activity was measured 48 h after transfection.

### *In vivo* Metastasis Assay

Female BABL/c nude mice (4–6 weeks old) were purchased from Shanghai SLAC Laboratory Animal Centre (Shanghai, China). For *in vivo* pulmonary metastasis assays, MKN-7 cells (1 × 10^7^) were transfected with agomir-NC (5 μM) or agomir-1224 (5 μM), respectively. The cells were injected into the lateral tail veins of each anesthetized nude mice (5 per group). Seven weeks after injection, the animals were killed, and lungs were embedded in paraffin and stained with HE for further pathological confirmation. The tumor metastasis nodules were obtained by the number of nodules on HE section with the aid of a dissecting microscope (OLYMPUS, Japan).

### Immunohistochemistry

Immunohistochemical staining was performed as we previously described (18). The staining intensity scores were divided into four groups: 0 (no staining), 1 (weak), 2 (moderate), or 3 (strong). The heterogeneity of staining was scored as 0 (≤5%), 1 (6–25%), 2 (26–50%), or 3 (>51%). After making the calculations, we evaluate FAK expression by determining the staining index with scores of 0, 1, 2, 3, 4, 6, or 9. Negative immunohistochemical expression is defined as the index ≤3 and positive expression was considered if the index was >4. Final scores were assigned by two independent pathologists.

### Statistics

All statistical analyses were carried out using the SPSS 17.0 software (SPSS, Chicago, IL) or R software (version 3.2.3). Continuous data are expressed as the means ± standard deviations (SD), and representative results were from three independent experiments. Statistical comparisons were calculated using Student's *t*-test. *P* < 0.05 was considered statistically significant. All experiments were performed at least in triplicates.

## Results

### miR-1224 Is Downregulated in Intestinal-Type GC and High miR-1224 Expression Was Associated With No Lymph-Node Metastasis and Good Prognosis

**Figure 1:**
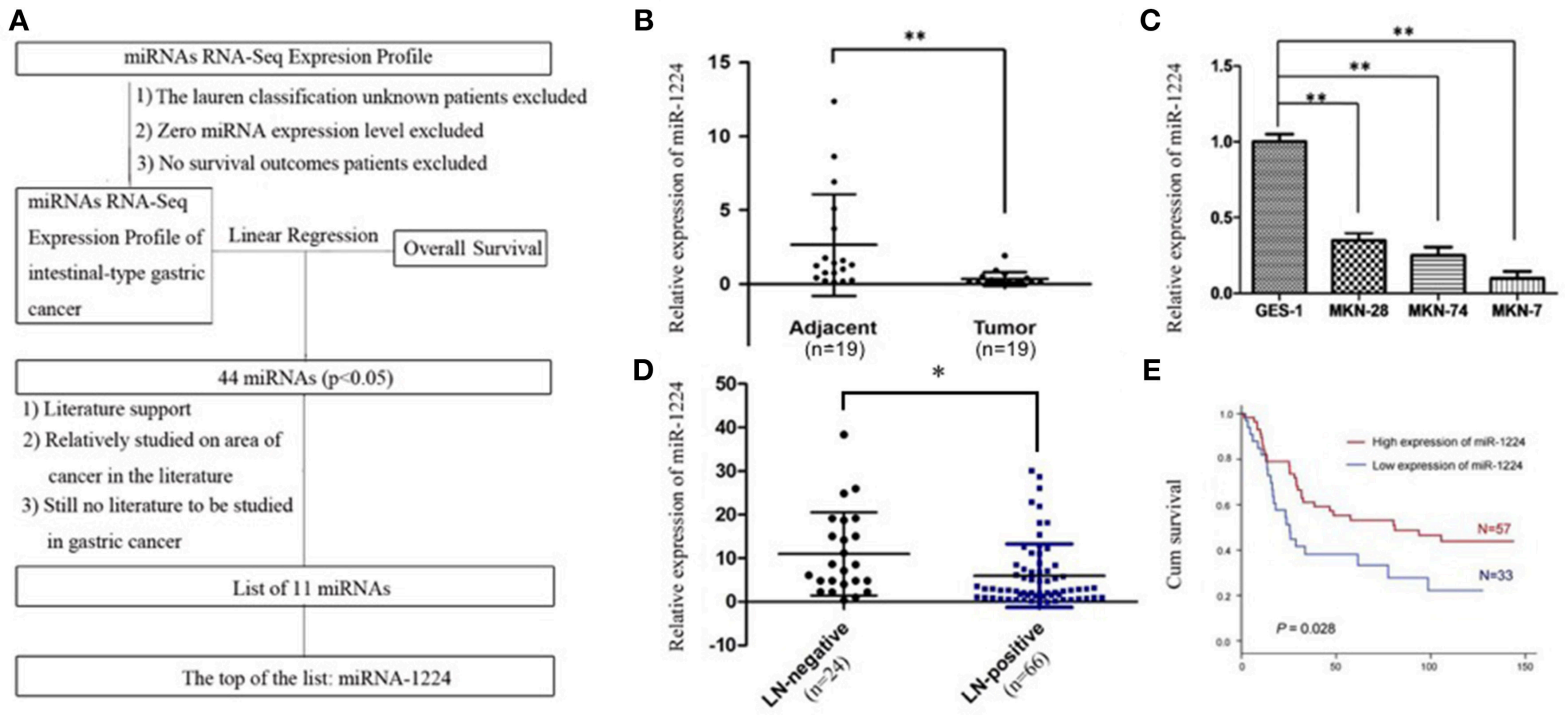
miR-1224 is downregulated in intestinal-type GC and high miR-1224 expression was associated with no lymph-node metastasis and good prognosis. **(A)** The flowchart of patient selection and study design in TCGA datasets. **(B)** The expression levels of miR-1224 in 19 pairs of GC tissues and the adjacent gastric cancer tissue, measured by qRT-PCR. The miRNA expression levels were normalized to the internal control U6. ^**^p < 0.01 vs. adjacent group. **(C)** Bar shows miR-1224 expression in three intestinal-type GC cell lines (MKN-28, MKN-7, MKN-74) and normal gastric mucous epithelium cell (GES-1). ^**^p < 0.01 vs.GES-1. **(D)** The expression levels of miR-1224 between 24 patients with no lymph-node metastasis (LN-negative) and 66 patients with lymph-node metastasis (LN-positive). ^*^p < 0.05 vs. LN-negative group. **(E)** Overall survival analysis shows that miR-1224 overexpression is associated with the good survival in 90 intestinal-type GC patients (*p* = 0.028). For **(B–D)**, data are presented as the mean ±SD.

Using TCGA datasets, we found that miR-1224 was the highest ranked candidate miRNA, whose expression levels were significantly correlated with patient survival in intestinal-type GC. The flowchart of patient selection and study design are shown in Figure 1A and Table S1.

The relative miR-1224 expression level was significantly decreased in 19 primary intestinal-type GC tumors compared to the paired normal mucosa (*p* < 0.01, Figure 1B). miR-1224 expression was also downregulated in three intestinal-type GC cell lines (MKN-28, MKN-7, MKN-74) compared to the normal gastric mucous epithelium cell line, GES-1 (*p* < 0.01, Figure 1C). We further validated the association between miR-1224 and clinicopathologic status in 90 intestinal-type GC patients (Table S2). As shown in Figure 1D and Table S2, there was a negative correlation between miR-1224 expression and lymph-node metastasis (*p* < 0.05) in 90 intestinal-type GC patients. Intestinal-type GC patients with high miR-1224 expression had a better survival outcome (*p* = 0.028, Figure 1E). Collectively, our results indicate that miR-1224 is downregulated in intestinal-type GC and high miR-1224 expression was associated with no lymph-node metastasis and good prognosis.

### miR-1224 Inhibits Cell Migration and EMT in Intestinal-Type GC Cells *in vitro*

**Figure 2:**
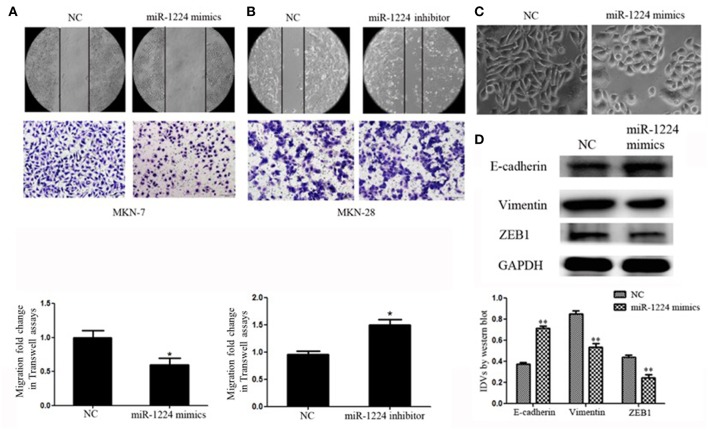
miR-1224 inhibits cell migration as well as EMT in intestinal-type GC *in vitro*. **(A)** The migratory abilities of MKN-7 cells transfected with miR-1224 mimics were detected by wound healing and Transwell assays. **(B)** The migratory abilities of MKN-28 cells transfected with miR-1224 inhibitor were detected by wound healing and Transwell assays. **(C)** The morphological changes of MKN-7 cells transfected with miR-1224 mimics in phase contrast microscopy, magnification ×200. **(D)** Western blot analysis of protein extracts from MKN-7 cells treated with miR-1224 mimics or NC. GAPDH was used as the endogenous control. IDVs represent the relative density values. For A, B and D, data are presented as the mean ±SD. ^*^p < 0.05 and ^**^p < 0.01 vs. NC group.

We measure miR-1224 expression among three intestinal-type GC cell lines, measured by qRT-PCR. MKN-7and MKN-28 cells exhibited the lowest and highest expression levels of miR-1224 in these intestinal-type GC lines, respectively (Figure 1B). Hence, we chose MKN-7 and MKN28 cell lines to perform the gain- or loss-of function. The transfection efficiencies of miR-1224 mimics and inhibitor in intestinal-type GC cells were shown in Figure S2. miR-1224-overexpression in MKN-7 cells significantly suppressed cell migration compared to the negative control (NC), shown in Figure 2A. Conversely, inhibition of miR-1224 caused less migration of MKN-28 cells (Figure 2B). These data suggest that miR-1224 could inhibit cell migration in intestinal-type GC cells.

We noticed that overexpression of miR-1224 promoted striking morphological changes in MKN-7 cells, which were hallmarks of reduced EMT (19–21). Specifically, the fibroblast, spindle-like morphology switched to the cobblestone-like appearance observed with epithelial cells (Figure 2C). The protein levels of EMT markers (E-cadherin, vimentin) and the transcription factor (finger E-box binding homeobox 1, ZEB1) were also markedly changed (Figure 2D). In contrast, miR-1224 inhibition caused dramatic morphological changes in MKN-28 cells from tight junctions to elongated and spindle-shaped mesenchymal cells along with decreased E-cadherin and increased vimentin and ZEB1 expression (Figure S3). Taken together, these data indicate that miR-1224 could inhibit the migration and EMT of intestinal-type GC cells *in vitro*.

### miR-1224 Inhibits Intestinal-Type GC Metastasis *in vivo*

**Figure 3:**
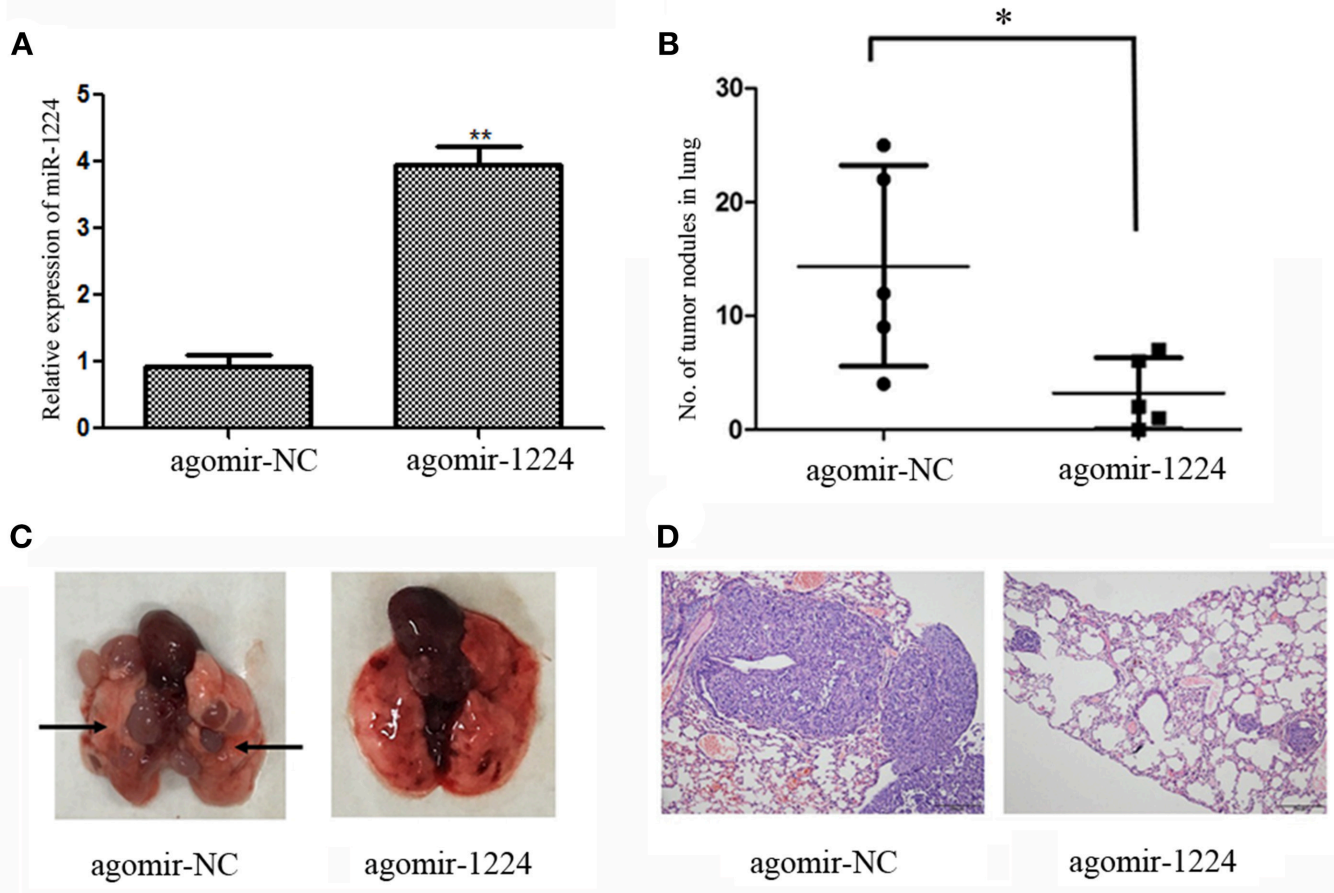
miR-1224 inhibits intestinal-type GC cell migration *in vivo*. **(A)** The expression levels of miR-1224 in the metastasis lung nodules of nude mice, measured by qRT-PCR. The nude mice injected with MKN-7 cells transfected with agomir-1224 (*n* = 5 per group) were compared with those injected with the MKN-7 cells transfected with agomir-NC. **(B)** The numbers of metastatic tumor colonies in the lungs of nude mice in respective group. Symbols represent individual mice. **(C)** Representative images of the lungs of nude mice. Black arrows show metastatic tumor colonies in the lung. **(D)** HE staining of metastatic tumor colonies in the lungs, magnification ×100. For A and B, data are presented as the mean ±SD. ^*^p < 0.05 and ^**^p < 0.01 vs. agomir-NC group.

We investigated the ability of miR-1224 to suppress intestinal-type GC lung metastasis *in vivo*. MKN-7 cells transfected with agomir-NC (5 μM) or agomir-1224 (5 μM) were injected intravenously into BALB/c nude mice through the lateral tail vein. The metastasis lung nodules from the agomir-1224 group showed a significant increase in the expression of miR-1224, compared with those from agomir-NC group (Figure 3A). The mice injected with agomir-1224 MKN-7 cells displayed a reduced number of metastatic lung nodules (*p* < 0.05), compared with those injected with agomir-NC cells (Figures 3B,C). The presence of metastatic foci in the lungs was confirmed by hematoxylin and eosin (HE) staining (Figure 3D). Therefore, these results suggest that miR-1224 could suppress the metastasis of intestinal-type GC cells *in vivo*.

### miR-1224 Directly Targets FAK

**Figure 4:**
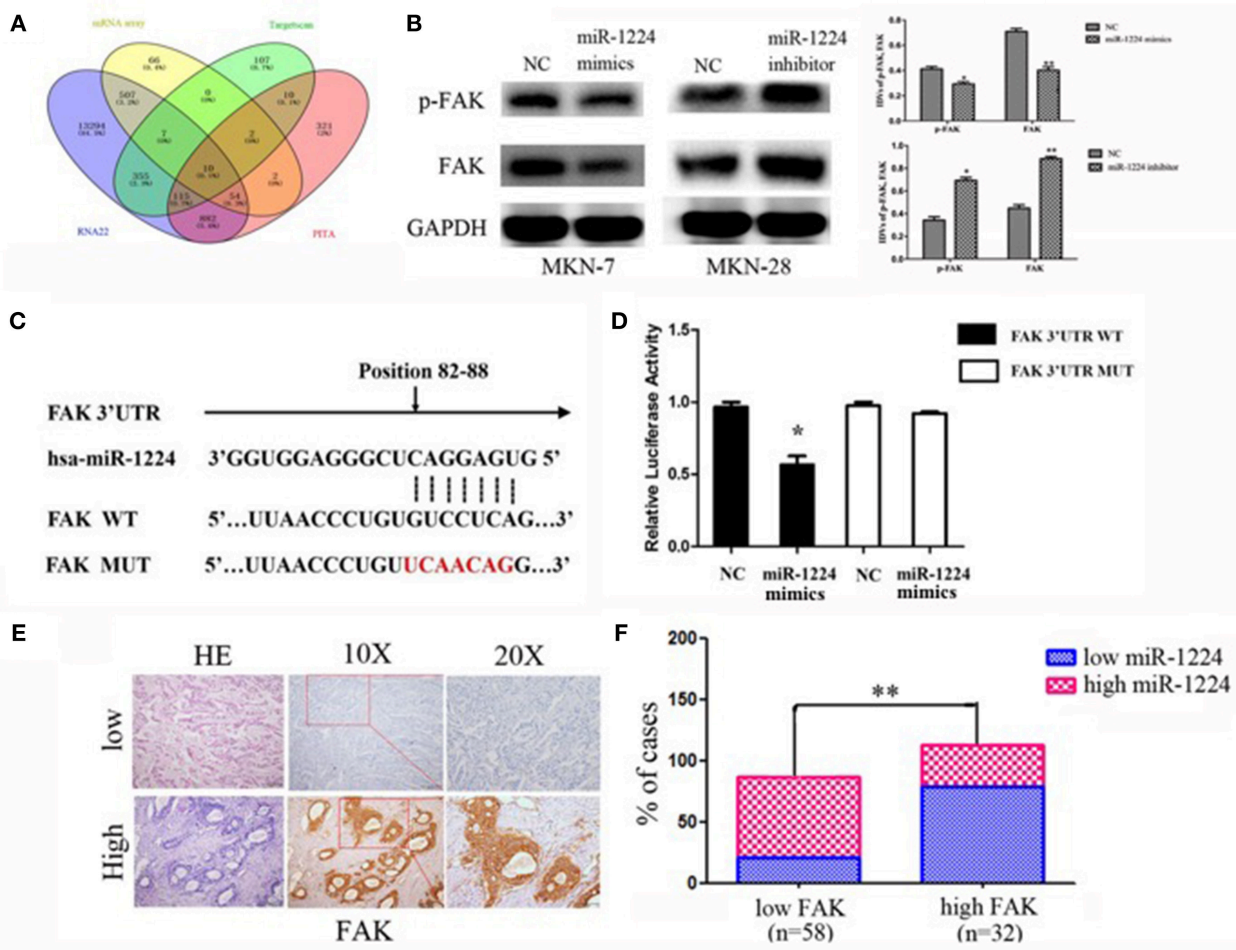
miR-1224 directly targets FAK. **(A)** The schematic diagram of screening strategy of target genes of miR-1224 using an mRNA array and three common miRNA target prediction engines (TargetScan, PicTar, and RNA22). **(B)** Western blot analysis of protein extracts from MKN-7 cells treated with miR-1224 mimics or NC, and MKN-28 cells treated with miR-1224 inhibitor or NC. GAPDH was used as the endogenous control. IDVs represent the relative density values. **(C)** Putative miR-1224 target sequence in wild-type (WT) and mutated (MUT) 3'UTR of FAK was generated as indicated. **(D)** Relative luciferase activity of FAK 3'UTR in the indicated cells co-transfected with the indicated reporters and miR-1224 mimics oligonucleotides. For B and D, data are presented as the mean ±SD. ^*^p < 0.05 and ^**^p < 0.01 vs. NC group. **(E)** FAK protein expression detected via IHC intestinal-type GC tissues. Top: low FAK expression; bottom: high FAK expression; left: HE stained sections, magnification ×100; middle: IHC-stained sections, magnification ×100; right: IHC-stained sections, magnification ×200. **(F)** Correlation between the expression of miR-1224 and FAK. Data are presented as the mean ±SD. ^**^p < 0.01 vs. low FAK group.

We used a mRNA array (Oebiotech Company) to screen for aberrant expression of mRNAs in miR-1224-overexpressing MKN-7 cells. The results identified 648 downregulated mRNAs (fold-change: >1.5, data not shown). The candidate genes were further screened using three common miRNA target prediction engines (TargetScan, PicTar, and RNA22), and then 10 mRNAs were selected (Figure 4A). KEGG pathway enrichment analysis were obtained for five of these mRNAs (Table S3), because of no results in other 5 mRNAs. Specifically, the analysis showed that FAK (also known as protein tyrosine kinase 2, PTK2) included the most critical metastasis promoting signaling pathway, which were frequently activated in GC (22, 23).

We found that FAK expression levels were decreased and increased after transfection of miR-1224 mimics or inhibitor, respectively, suggesting that FAK expression was regulated by miR-1224 (Figure 4B). In addition, we generated luciferase reporter constructs that contained the predicted binding site for miR-1224, which was observed in the 3′UTR of FAK, or the corresponding mutant binding site (position 82 to 88) (Figure 4C). Overexpression of miR-1224 significantly reduced the relative luciferase activity of the wild-type construct compared to the control cells (*p* < 0.05). In contrast, miR-1224 did not affect the relative luciferase activity of the mutant construct (*p* = 0.194, Figure 4D). Moreover, we examined FAK expression in intestinal-type GC using immunohistochemistry (Figure 4E). miR-1224 was significantly increased in low FAK group patients (χ^2^ = 17.931, *p* < 0.001) (Figure 4F). Collectively, these results demonstrated that miR-1224 could directly target the 3′-UTR of FAK.

### The miR-1224-FAK Axis Suppresses Intestinal-Type GC Cell Migration and EMT by Inhibiting the STAT3 and NF-κB Pathways

**Figure 5:**
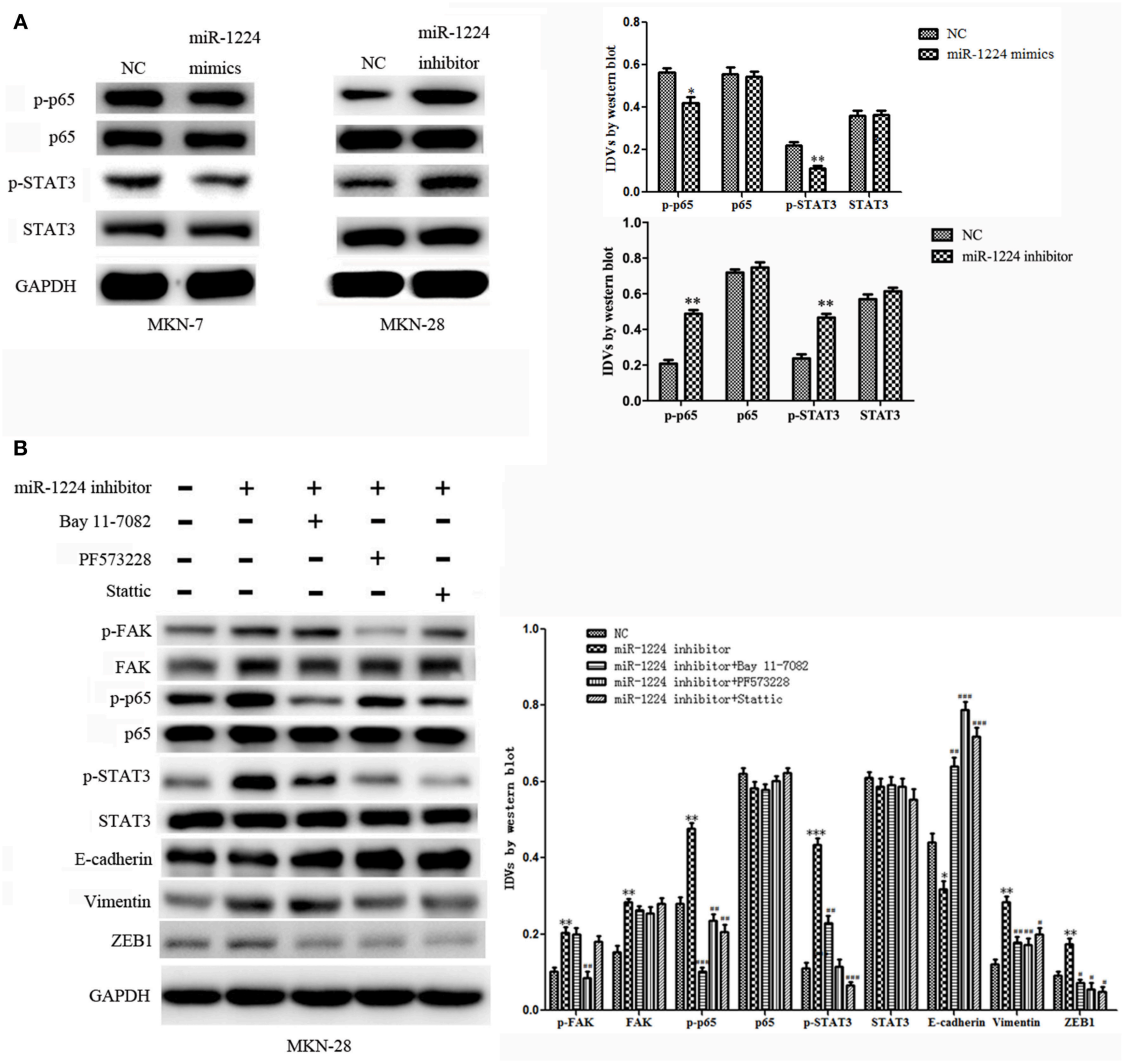
The miR-1224-FAK axis suppresses intestinal-type cell migration and EMT by inhibiting the STAT3 and NF-κB pathways. **(A)** Western blot analysis of protein extracts from MKN-7 cells treated with miR-1224 mimics or NC, and MKN-28 cells treated with miR-1224 inhibitor or NC. **(B)** Western blot analysis of protein extracts from MKN-28 cells treated with miR-1224 inhibitor or NC, and MKN-28 cells treated with combination of miR-1224 inhibitor and Bay 11-7082 (NF-κB inhibitor), or PF573228(FAK inhibitor), or Stattic (STAT3 inhibitor). For **(A,B)**, data are presented as the mean ± SD. ^*^p < 0.05, ^**^p < 0.01 and ^***^p < 0.001 vs. NC group. ^#^*p* < 0.05, ^*##*^*p* < 0.01 and ^*###*^*p* < 0.001 vs. miR-1224 inhibitor group. GAPDH was used as the endogenous control. IDVs represent the relative density values.

To explore the mechanism of miR-1224-mediated regulation of FAK expression, Western blot analysis was performed to determine the effects of miR-1224 overexpression or miR-1224 knockdown on the STAT3 and NF-κB pathways. We observed reduced levels of phosphorylated STAT3 and phosphorylated P65 in MKN-7 following miR-1224 overexpression and increased phosphorylation of these proteins following miR-1224 knockdown in MKN-28 cells. However, changes in miR-1224 levels did not alter the levels of total STAT3 and P65 protein (Figure 5A). These results suggest that miR-1224 could inhibit the STAT3 and NF-κB pathways in intestinal-type GC cells.

Previous studies have shown that silencing of FAK expression inhibited migration and EMT in tumor cells (24–32), and the STAT3 and NF-κB signaling pathways have been shown to play an important role in the metastasis of GC and EMT (33–35). Therefore, we investigated whether the miR-1224-FAK axis regulated the STAT3 and NF-κB signaling pathways in intestinal-type GC. Western blot analysis demonstrated that PF573228 (FAK inhibitor) inhibited the activation of FAK, NF-κB, and STAT3 pathways induced by miR-1224 knockdown. The inhibition of the NF-κB pathway by Bay 11-7082 abrogated the effects of miR-1224 knockdown on the NF-κB pathway. Interestingly, the activity of the STAT3 pathway was also partially suppressed, but no changes were observed in the FAK pathway activity. Similarly, the inhibition of the STAT3 pathway by Stattic abolished the activation the of the STAT3 pathway induced by miR-1224 knockdown. The activity of the NF-κB pathway was also partially suppressed in the absence of changes in FAK pathway activity. Importantly, the expression of all the EMT-related markers, including E-cadherin, vimentin, and ZEB1, was restored in the presence of these three pathway inhibitors (Figure 5B). Taken together, our data suggest that miR-1224 exerts its effects through FAK-mediated dysregulation of the STAT3 and NF-κB signaling pathways, and there is a mutual regulatory mechanism between these two pathways.

### Repression of FAK Is Required for the miR-1224-Mediated Inhibition of Cell Migration in Intestinal-Type GC

**Figure 6:**
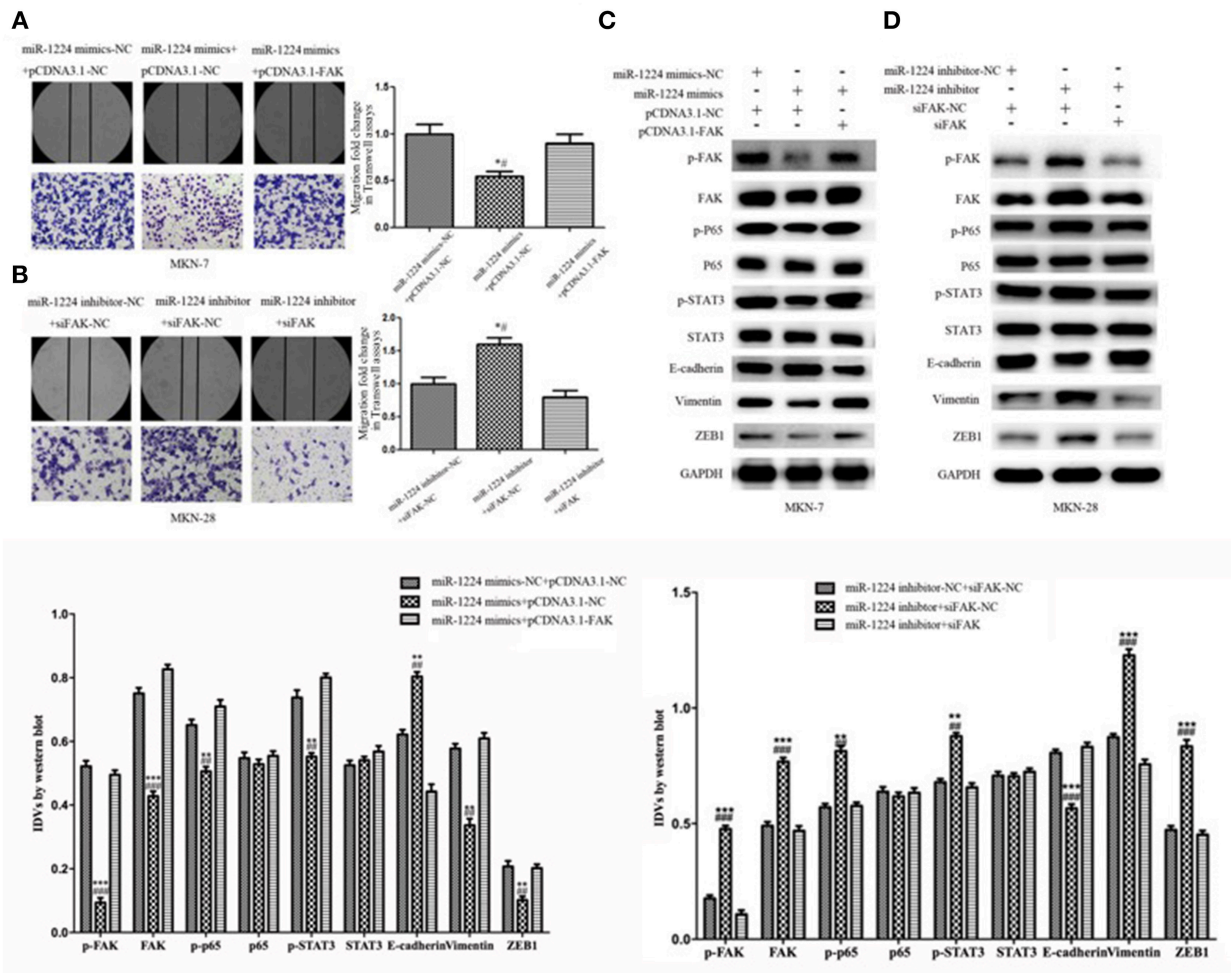
Repression of FAK is required for the miR-1224-mediated inhibition of cell migration in intestinal-type GC. **(A)** The migratory abilities of MKN-7 cells transfected with combination of miR-1224 mimics-NC and pcDNA3.1-NC, or miR-1224 mimics and pcDNA3.1-NC, or miR-1224 mimics and pcDNA3.1-FAK were detected by wound healing and Transwell assays. ^*^p < 0.05 vs. miR-1224 mimics-NC +pcDNA3.1-NC group. ^#^*p* < 0.05 vs. miR-1224 mimics +pcDNA3.1-FAK group. **(B)** The migratory abilities of MKN-28 cells transfected with combination of miR-1224 inhibitor-NC and siFAK-NC, or miR-1224 inhibitor and siFAK-NC, or miR-1224 inhibitor and si-FAK were detected by wound healing and Transwell assays. ^*^p < 0.05 vs. miR-1224 inhibitor-NC + siFAK-NC group. ^#^*p* < 0.05 vs. miR-1224 inhibitor +si-FAK group. **(C)** Western blot analysis of protein extracts from MKN-7 cells treated with combination of miR-1224 mimics-NC and pcDNA3.1-NC, or miR-1224 mimics and pcDNA3.1-NC, or miR-1224 mimics and pcDNA3.1-FAK. ^**^p < 0.01 and ^***^p < 0.001 vs. miR-1224 mimics-NC +pcDNA3.1-NC group. ^##^*p* < 0.01 and ^###^*p* < 0.001 vs. miR-1224 mimics +pcDNA3.1-FAK group. **(D)** Western blot analysis of protein extracts from MKN-28 cells treated with combination of miR-1224 inhibitor-NC and siFAK-NC, or miR-1224 inhibitor and siFAK-NC, or miR-1224 inhibitor and si-FAK. ^**^p < 0.01 and ^***^p < 0.001 vs. miR-1224 inhibitor-NC + siFAK-NC group. ^##^*p* < 0.01 and ^###^*p* < 0.001 vs. miR-1224 inhibitor +si-FAK group. For C and D, GAPDH was used as the endogenous control. IDVs represent the relative density values. For A-D, data are presented as the mean ± SD.

**Figure 7:**
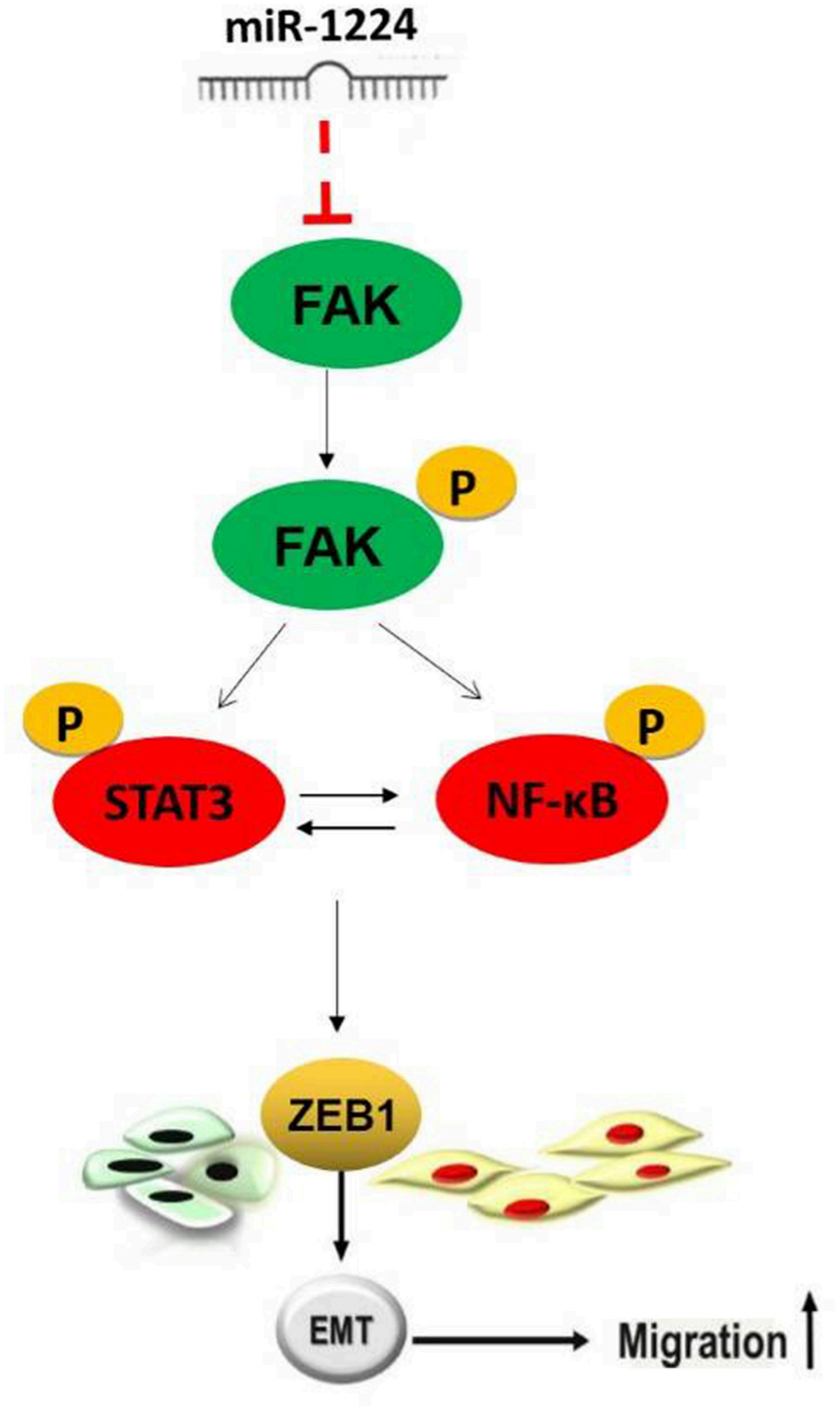
Schematic representation of the proposed model.

Restoration of FAK expression in miR-1224-overexpressing MKN-7 cells significantly increased cell migration as demonstrated by both the wound healing assays and Transwell assays (Figure 6A). Furthermore, FAK and EMT marker expression were rescued by the co-transfection of a FAK-overexpression vector and miR-1224 mimics in MKN-7 cells (Figure 6C). Furthermore, FAK silencing partially abolished the effects of miR-1224 knockdown in MKN-28 cells (Figures 6B,D). Together, these data suggest that miR-1224 inhibited the metastasis of intestinal-type GC by suppressing the FAK-mediated activation of the STAT3 and NF-κB pathways, and subsequent EMT (Figure 7).

## Discussion

Although there have been multiple studies about the roles of miRNAs in GC, most studies have focused on GC in general and not on specific types of GC. Even in the analysis of the Lauren subtype of GC, there have been few miRNAs studies for intestinal-type GC. Azarbarzin et al. reported that miR-383 was downregulated in 40 pairs of intestinal-type GC tissues compared to paired non-tumor tissue (36). To our knowledge, no prognostic analysis of miRNAs in the Lauren subtype of GC is available.

Previous studies have shown that miR-1224 may be downregulated or upregulated depending on the type of cancer. For instance, miR-1224 is downregulated in glioma, lung cancer, and colorectal cancer, but upregulated in bladder cancer (37–42). Moreover, miR-1224 expression is correlated with good survival in glioma and colon cancer (40–42). It is significantly upregulated in patients with a complete response to neoadjuvant chemoradiotherapy in locally advanced rectal cancer (37). In addition to its role in cancer, miR-1224 is associated with tube-formation of human primary endothelial cells, the occurrence and development of keloids, and cell proliferation in acute liver failure (43–45). In the present study, we identified miR-1224 as a potential survival-related miRNA in intestinal-type GC. Furthermore, miR-1224 showed no statistically significant prognosis differences in diffuse-type GC patients by TCGA analysis (data not shown), thus, it may have an important prognostic value in intestinal-type GC. Downregulation of miR-1224 expression could promote intestinal-type GC cell migration and EMT *in vitro* and *in vivo*. Thus, miR-1224 appears to have a tumor-suppressive function in intestinal-type GC.

Although mechanisms for miR-1224 activity in VEGF signaling and the repression of NOTCH signaling and the TGF-β1/Smad3 signaling pathway have been reported (44, 45), the role of miR-1224 in tumor biogenesis is unclear. Currently, only one report has demonstrated that miR-1224 inhibited tumor-associated activity in malignant gliomas by targeting cAMP response element-binding protein (CREB1) (42). In this study, we combined the analyses of a mRNA array and bioinformatics database to identify candidate target genes for miR-1224, and the major target gene identified was FAK. FAK is a protein tyrosine kinase that is overexpressed in several cancers and promotes cancer progression and metastasis (46). The de-adhesion of tumor cells from their primary site has been proposed as the initial step of metastasis (47). FAK is thought to be an important regulatory element in tumor cell adhesion and migration (30). In addition, FAK may play a crucial role in GC metastasis (23, 48). However, the mechanisms mediating the role of FAK in GC metastasis remain complex and undefined. Our finding demonstrated that ectopic expression of FAK in miR-1224 overexpressing cells can rescue miR-1224-mediated inhibition of intestinal-type GC cell migration. Numerous studies have shown that activation of the STAT3 and NF-κB pathways is essential for the EMT and metastasis (33–35). Our results demonstrated that the antitumor effect of miR-1224 is mediated by the inhibition of FAK and downstream STAT3 and NF-κB pathways in intestinal-type GC cell with a mutual regulatory mechanism shared between these two pathways.

We demonstrated that miR-1224 is downregulated in intestinal-type GC and miR-1224 inhibited the metastasis of intestinal-type GC by suppressing the FAK-mediated activation of the STAT3 and NF-κB pathways, and subsequent EMT. These results provide a better understanding of the molecular mechanisms of intestinal-type GC and suggest that miR-1224 may serve as a novel biomarker and effective therapeutic approach to inhibit intestinal-type GC.

## Data Availability

Publicly available datasets were analyzed in this study. This data can be found here: https://www.cancer.gov/about-nci/organization/ccg/research/structural-genomics/tcga.

## Ethics Statement

The study was approved by the Ethics Committee of the First Hospital of China Medical University with written informed consent from all subjects. All subjects gave written informed consent in accordance with the Declaration of Helsinki. The animal studies were used under conditions approved by the Institutional Animal Care and Use Committee of China Medical University.

## Author Contributions

TW, XC, XQ, and YL contributed to conceptualization. ZL contributed to data curation and formal analysis. TW, XQ, and YL contributed to funding acquisition. JW contributed to investigation and writing the original draft. JW and LG contributed to methodology. JW, TW, ZL, LG, and LH contributed to project administration. XY, JZ, and HT contributed to resources. ZL contributed to software. TW, XC, XQ, and YL contributed to supervision. ZL and LG contributed to validation. JW and LH contributed to visualization. TW, XQ, and YL contributed to writing, editing and review.

### Conflict of Interest Statement

The authors declare that the research was conducted in the absence of any commercial or financial relationships that could be construed as a potential conflict of interest.

## Supplementary Material

Figure S1 The transfection efficiency of the siRNA against FAK. **(A)** The transfection efficiency of the siRNA against FAK in MKN-7 cells, measured by Western blot analysis. **(B)** The transfection efficiency of efficiency of the siRNA against FAK in MKN-28 cells, measured by Western blot analysis. For **A** and **B**, data are presented as the mean ±SD. ^*^*p* < 0.05 and ^**^*p* < 0.01 vs. NC group. GAPDH was used as the endogenous control. IDVs represent the relative density values.

Figure S2 The transfection efficiencies of miR-1224 mimics and inhibitor in intestinal-type GC cells. **(A)** The transfection efficiency of MKN-7 cells treated with miR-1224 mimics or NC, measured by qRT-PCR. **(B)** The transfection efficiency of MKN-28 cells treated with miR-1224 inhibitor or NC, measured by qRT-PCR. For **A** and **B**, data are presented as the mean ±SD. ^**^*p* < 0.01 and ^***^*p* < 0.001 vs. NC group.

Figure S3 miR-1224 inhibition caused dramatic morphological and Western blot changes in MKN-28 cells, **(A)** The morphological changes of MKN-28 cells transfected with miR-1224 inhibitor in phase contrast microscopy, magnification ×200. **(B)** Western blot analysis of protein extracts from MKN-28 cells treated with miR-1224 inhibitor or NC. For **A** and **B**, data are presented as the mean ±SD. ^*^*p* < 0.05 and ^**^*p* < 0.01 vs. NC group. GAPDH was used as the endogenous control. IDVs represent the relative density values.

Table S1 11 candidate miRNAs correlated with patient survival in intestinal-type GC in TCGA datasets.

Table S2 The correlations of miR-1224 with clinicopathological features of intestinal-type GC.

Table S3 KEGG pathway results of candidate target genes.

## References

[1] Chen W Zheng R Baade PD Zhang S Zeng H Bray F. Cancer statistics in China, 2015. CA Cancer J Clin. (2016) 66:115–32. 10.3322/caac.21338 26808342

[2] Jemal A Bray F Center MM Ferlay J Ward E Forman D. Global cancer statistics. CA Cancer J Clin. (2011) 61:69–90. 10.3322/caac.20107 21296855

[3] Kitano S Shiraishi N Uyama I Sugihara K Tanigawa N Japanese Laparoscopic Surgery Study G. A multicenter study on oncologic outcome of laparoscopic gastrectomy for early cancer in Japan. Ann Surg. (2007) 245:68–72. 10.1097/01.sla.0000225364.03133.f8 17197967 PMC1867926

[4] Uedo N Takeuchi Y Ishihara R. Endoscopic management of early gastric cancer: endoscopic mucosal resection or endoscopic submucosal dissection: data from a Japanese high-volume center and literature review. Ann Gastroenterol. (2012) 25:281–90. 24714247 PMC3959406

[5] Lauren P. The Two histological main types of gastric carcinoma: diffuse and so-called intestinal-type carcinoma. an attempt at a histo-clinical Classification. Acta Pathol Microbiol Scand. (1965) 64:31–49. 14320675 10.1111/apm.1965.64.1.31

[6] Ni P Xu H Xue H Lin B Lu Y. A meta-analysis of interleukin-10-1082 promoter polymorphism associated with gastric cancer risk. DNA Cell Biol. (2012) 31:582–91. 10.1089/dna.2011.1440 22335769 PMC3322401

[7] Qiu MZ Li Q Wang ZQ Liu TS Liu Q Wei XL. HER2-positive patients receiving trastuzumab treatment have a comparable prognosis with HER2-negative advanced gastric cancer patients: a prospective cohort observation. Int J Cancer. (2014) 134:2468–77. 10.1002/ijc.28559 24155030

[8] Tsuchiya T Tamura G Sato K Endoh Y Sakata K Jin Z. Distinct methylation patterns of two APC gene promoters in normal and cancerous gastric epithelia. Oncogene. (2000) 19:3642–6. 10.1038/sj.onc.1203704 10951570

[9] Xue H Lin B Ni P Xu H Huang G. Interleukin-1B and interleukin-1 RN polymorphisms and gastric carcinoma risk: a meta-analysis. J Gastroenterol Hepatol. (2010) 25:1604–17. 10.1111/j.1440-1746.2010.06428.x 20880168

[10] Albulescu R Neagu M Albulescu L Tanase C. Tissular and soluble miRNAs for diagnostic and therapy improvement in digestive tract cancers. Expert Rev Mol Diagn. (2011) 11:101–20. 10.1586/erm.10.106 21171925

[11] Lu J Getz G Miska EA Alvarez-Saavedra E Lamb J Peck D. MicroRNA expression profiles classify human cancers. Nature. (2005) 435:834–8. 10.1038/nature03702 15944708

[12] Bandres E Bitarte N Arias F Agorreta J Fortes P Agirre X. microRNA-451 regulates macrophage migration inhibitory factor production and proliferation of gastrointestinal cancer cells. Clin Cancer Res. (2009) 15:2281–90. 10.1158/1078-0432.CCR-08-1818 19318487

[13] Brenner B Hoshen MB Purim O David MB Ashkenazi K Marshak G. MicroRNAs as a potential prognostic factor in gastric cancer. World J Gastroenterol. (2011) 17:3976–85. 10.3748/wjg.v17.i35.3976 22046085 PMC3199555

[14] Li X Zhang Y Zhang Y Ding J Wu K Fan D. Survival prediction of gastric cancer by a seven-microRNA signature. Gut. (2010) 59:579–85. 10.1136/gut.2008.175497 19951901

[15] Nishida N Mimori K Fabbri M Yokobori T Sudo T Tanaka F. MicroRNA-125a-5p is an independent prognostic factor in gastric cancer and inhibits the proliferation of human gastric cancer cells in combination with trastuzumab. Clin Cancer Res. (2011) 17:2725–33. 10.1158/1078-0432.CCR-10-2132 21220473

[16] Tsai KW Wu CW Hu LY Li SC Liao YL Lai CH. Epigenetic regulation of miR-34b and miR-129 expression in gastric cancer. Int J Cancer. (2011) 129:2600–10. 10.1002/ijc.25919 21960261

[17] Xu L Zhang Y Liu J Qu J Hu X Zhang F. TRAIL-activated EGFR by Cbl-b-regulated EGFR redistribution in lipid rafts antagonises TRAIL-induced apoptosis in gastric cancer cells. Eur J Cancer. (2012) 48:3288–99. 10.1016/j.ejca.2012.03.005 22456178

[18] Li H Xu L Li C Zhao L Ma Y Zheng H. Ubiquitin ligase Cbl-b represses IGF-I-induced epithelial mesenchymal transition via ZEB2 and microRNA-200c regulation in gastric cancer cells. Mol Cancer. (2014) 13:136. 10.1186/1476-4598-13-136 24885194 PMC4052283

[19] Dykxhoorn DM. MicroRNAs and metastasis: little RNAs go a long way. Cancer Res. (2010) 70:6401–6. 10.1158/0008-5472.CAN-10-1346 20663901 PMC2922433

[20] Hao J Zhang Y Deng M Ye R Zhao S Wang Y. MicroRNA control of epithelial-mesenchymal transition in cancer stem cells. Int J Cancer. (2014) 135:1019–27. 10.1002/ijc.28761 24500893

[21] Katoh M. Epithelial-mesenchymal transition in gastric cancer (review). Int J Oncol. (2005) 27:1677–83. 10.3892/ijo.27.6.1677 16273224

[22] Gan L Meng J Xu M Liu M Qi Y Tan C. Extracellular matrix protein 1 promotes cell metastasis and glucose metabolism by inducing integrin beta4/FAK/SOX2/HIF-1alpha signaling pathway in gastric cancer. Oncogene. (2018) 37:744–55. 10.1038/onc.2017.363 29059156

[23] Guo LL He ZC Yang CQ Qiao PT Yin GL. Epigenetic silencing of olfactomedin-4 enhances gastric cancer cell invasion via activation of focal adhesion kinase signaling. BMB Rep. (2015) 48:630–5. 10.5483/BMBRep.2015.48.11.130 26303970 PMC4911205

[24] Bailey KM Liu J. Caveolin-1 up-regulation during epithelial to mesenchymal transition is mediated by focal adhesion kinase. J Biol Chem. (2008) 283:13714–24. 10.1074/jbc.M709329200 18332144 PMC2376249

[25] Cai L Ye Y Jiang Q Chen Y Lyu X Li J. Epstein-Barr virus-encoded microRNA BART1 induces tumour metastasis by regulating PTEN-dependent pathways in nasopharyngeal carcinoma. Nat Commun. (2015) 6:7353. 10.1038/ncomms8353 26135619 PMC4507016

[26] Chen JS Li HS Huang JQ Dong SH Huang ZJ Yi W. MicroRNA-379-5p inhibits tumor invasion and metastasis by targeting FAK/AKT signaling in hepatocellular carcinoma. Cancer Lett. (2016) 375:73–83. 10.1016/j.canlet.2016.02.043 26944318

[27] Cicchini C Laudadio I Citarella F Corazzari M Steindler C Conigliaro A. TGFbeta-induced EMT requires focal adhesion kinase (FAK) signaling. Exp Cell Res. (2008) 314:143–52. 10.1016/j.yexcr.2007.09.005 17949712

[28] Fan H Zhao X Sun S Luo M Guan JL. Function of focal adhesion kinase scaffolding to mediate endophilin A2 phosphorylation promotes epithelial-mesenchymal transition and mammary cancer stem cell activities *in vivo*. J Biol Chem. (2013) 288:3322–33. 10.1074/jbc.M112.420497 23255596 PMC3561552

[29] Jean C Chen XL Nam JO Tancioni I Uryu S Lawson C. Inhibition of endothelial FAK activity prevents tumor metastasis by enhancing barrier function. J Cell Biol. (2014) 204:247–63. 10.1083/jcb.201307067 24446483 PMC3897185

[30] Kong X Li G Yuan Y He Y Wu X Zhang W. MicroRNA-7 inhibits epithelial-to-mesenchymal transition and metastasis of breast cancer cells via targeting FAK expression. PLoS ONE. (2012) 7:e41523. 10.1371/journal.pone.0041523 22876288 PMC3410899

[31] Li XY Zhou X Rowe RG Hu Y Schlaepfer DD Ilic D. Snail1 controls epithelial-mesenchymal lineage commitment in focal adhesion kinase-null embryonic cells. J Cell Biol. (2011) 195:729–38. 10.1083/jcb.201105103 22105351 PMC3257570

[32] Tavora B Reynolds LE Batista S Demircioglu F Fernandez I Lechertier T. Endothelial-cell FAK targeting sensitizes tumours to DNA-damaging therapy. Nature. (2014) 514:112–6. 10.1038/nature13541 25079333 PMC4533916

[33] Cheng Y Song Y Qu J Che X Song N Fan Y. The chemokine receptor CXCR4 and c-MET cooperatively promote epithelial-mesenchymal transition in gastric cancer cells. Transl Oncol. (2018) 11:487–97. 10.1016/j.tranon.2018.02.002 29494948 PMC5884220

[34] Hou J Wang T Xie Q Deng W Yang JY Zhang SQ. N-Myc-interacting protein (NMI) negatively regulates epithelial-mesenchymal transition by inhibiting the acetylation of NF-kappaB/p65. Cancer Lett. (2016) 376:22–33. 10.1016/j.canlet.2016.02.015 27012186

[35] Xu L Zhou R Yuan L Wang S Li X Ma H. IGF1/IGF1R/STAT3 signaling-inducible IFITM2 promotes gastric cancer growth and metastasis. Cancer Lett. (2017) 393:76–85. 10.1016/j.canlet.2017.02.014 28223169

[36] Azarbarzin S Feizi MAH Safaralizadeh R Kazemzadeh M Fateh A. The Value of MiR-383, an Intronic MiRNA, as a Diagnostic and Prognostic Biomarker in Intestinal-Type Gastric Cancer. Biochem Genet. (2017) 55:244–52. 10.1007/s10528-017-9793-x 28243881

[37] Della Vittoria Scarpati G Falcetta F Carlomagno C Ubezio P Marchini S De Stefano A A specific miRNA signature correlates with complete pathological response to neoadjuvant chemoradiotherapy in locally advanced rectal cancer. Int J Radiat Oncol Biol Phys. (2012) 83:1113–9. 10.1016/j.ijrobp.2011.09.030 22172905

[38] Dudziec E Miah S Choudhry HM Owen HC Blizard S Glover M. Hypermethylation of CpG islands and shores around specific microRNAs and mirtrons is associated with the phenotype and presence of bladder cancer. Clin Cancer Res. (2011) 17:1287–96. 10.1158/1078-0432.CCR-10-2017 21138856

[39] Miah S Dudziec E Drayton RM Zlotta AR Morgan SL Rosario DJ. An evaluation of urinary microRNA reveals a high sensitivity for bladder cancer. Br J Cancer. (2012) 107:123–8. 10.1038/bjc.2012.221 22644299 PMC3389418

[40] Mosakhani N Lahti L Borze I Karjalainen-Lindsberg ML Sundstrom J Ristamaki R. MicroRNA profiling predicts survival in anti-EGFR treated chemorefractory metastatic colorectal cancer patients with wild-type KRAS and BRAF. Cancer Genet. (2012) 205:545–51. 10.1016/j.cancergen.2012.08.003 23098991

[41] Nymark P Guled M Borze I Faisal A Lahti L Salmenkivi K. Integrative analysis of microRNA, mRNA and aCGH data reveals asbestos- and histology-related changes in lung cancer. Genes Chromosomes Cancer. (2011) 50:585–97. 10.1002/gcc.20880 21563230

[42] Qian J Li R Wang YY Shi Y Luan WK Tao T. MiR-1224-5p acts as a tumor suppressor by targeting CREB1 in malignant gliomas. Mol Cell Biochem. (2015) 403:33–41. 10.1007/s11010-015-2334-1 25648114

[43] Roy S Bantel H Wandrer F Schneider AT Gautheron J Vucur M. miR-1224 inhibits cell proliferation in acute liver failure by targeting the antiapoptotic gene Nfib. J Hepatol. (2017) 67:966–78. 10.1016/j.jhep.2017.06.007 28645739

[44] Sakai E Miura Y Suzuki-Kouyama E Oka K Tachibana M Kawabata K. A mammalian mirtron miR-1224 promotes tube-formation of human primary endothelial cells by targeting anti-angiogenic factor epsin2. Sci Rep. (2017) 7:5541. 10.1038/s41598-017-05782-3 28717225 PMC5514154

[45] Yao X Cui X Wu X Xu P Zhu W Chen X. Tumor suppressive role of miR-1224-5p in keloid proliferation, apoptosis and invasion via the TGF-beta1/Smad3 signaling pathway. Biochem Biophys Res Commun. (2018) 495:713–20. 10.1016/j.bbrc.2017.10.070 29050938

[46] Sulzmaier FJ Jean C Schlaepfer DD. FAK in cancer: mechanistic findings and clinical applications. Nat Rev Cancer. (2014) 14:598–610. 10.1038/nrc3792 25098269 PMC4365862

[47] Thiery JP. Epithelial-mesenchymal transitions in tumour progression. Nat Rev Cancer. (2002) 2:442–54. 10.1038/nrc822 12189386

[48] Du T Qu Y Li J Li H Su L Zhou Q. Maternal embryonic leucine zipper kinase enhances gastric cancer progression via the FAK/Paxillin pathway. Mol Cancer. (2014) 13:100. 10.1186/1476-4598-13-100 24885567 PMC4113179

