# Supplementary figures and images for: MicroRNA-1224 Inhibits Tumor Metastasis in Intestinal-Type Gastric Cancer by Directly Targeting FAK
Source: Front Oncol. 2019 Apr 4;9:222. doi: 10.3389/fonc.2019.00222 (PMC6458237; doi:10.3389/fonc.2019.00222)

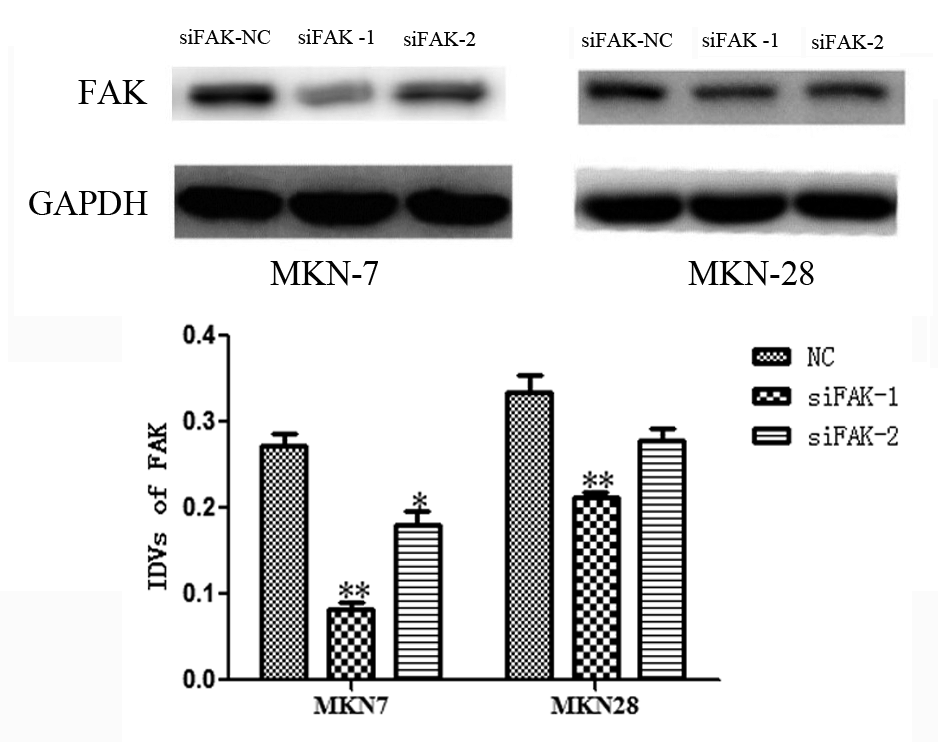

Supplement: Figure S1 — The transfection efficiency of the siRNA against FAK. (A) The transfection efficiency of the siRNA against FAK in MKN-7 cells, measured by Western blot analysis. (B) The transfection efficiency of efficiency of the siRNA against FAK in MKN-28 cells, measured by Western blot analysis. For A and B, data are presented as the mean ±SD. *p < 0.05 and **p < 0.01 vs. NC group. GAPDH was used as the endogenous control. IDVs represent the relative density values. [file Image_1.TIF]

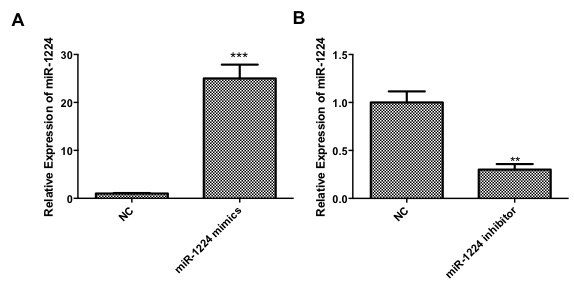

Supplement: Figure S2 — The transfection efficiencies of miR-1224 mimics and inhibitor in intestinal-type GC cells. (A) The transfection efficiency of MKN-7 cells treated with miR-1224 mimics or NC, measured by qRT-PCR. (B) The transfection efficiency of MKN-28 cells treated with miR-1224 inhibitor or NC, measured by qRT-PCR. For A and B, data are presented as the mean ±SD. **p < 0.01 and ***p < 0.001 vs. NC group. [file Image_2.JPEG]

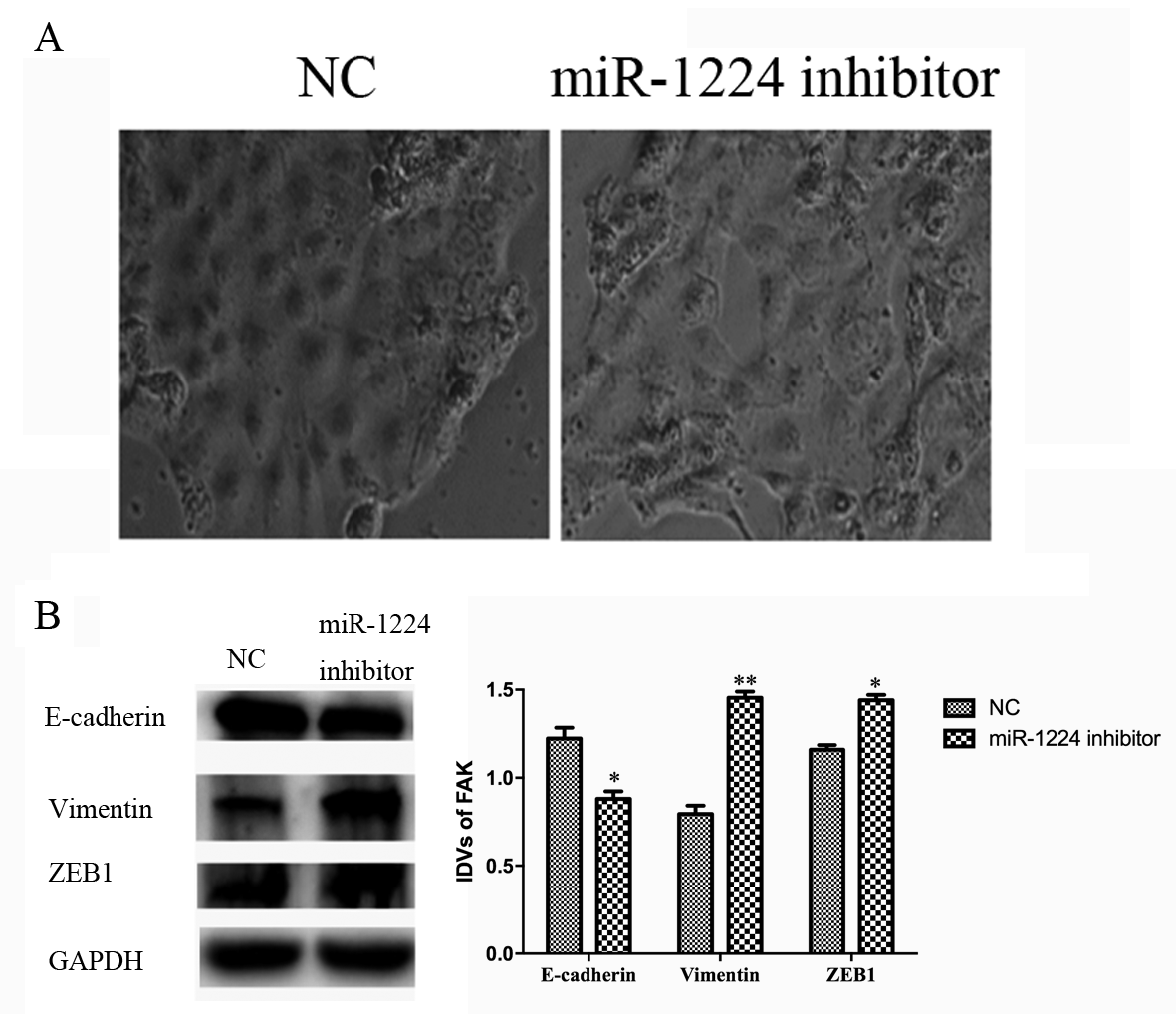

Supplement: Figure S3 — miR-1224 inhibition caused dramatic morphological and Western blot changes in MKN-28 cells, (A) The morphological changes of MKN-28 cells transfected with miR-1224 inhibitor in phase contrast microscopy, magnification ×200. (B) Western blot analysis of protein extracts from MKN-28 cells treated with miR-1224 inhibitor or NC. For A and B, data are presented as the mean ±SD. *p < 0.05 and **p < 0.01 vs. NC group. GAPDH was used as the endogenous control. IDVs represent the relative density values. [file Image_3.TIF]
