# Supplementary material for: MicroRNA-1224 Inhibits Tumor Metastasis in Intestinal-Type Gastric Cancer by Directly Targeting FAK
Source: Front Oncol. 2019 Apr 4;9:222. doi: 10.3389/fonc.2019.00222 (PMC6458237; doi:10.3389/fonc.2019.00222)
Supplement: Table S1 — 11 candidate miRNAs correlated with patient survival in intestinal-type GC in TCGA datasets. [file Table_1.DOCX]

Table S1.11 candidate miRNAs correlated with patient survival in intestinal-type GC in TCGA datasets

| Rank | miRNAs | HR | P value |
| --- | --- | --- | --- |
| 1 | mir-1224 | 0.494 | 0.01 |
| 2 | mir-592 | 0.53 | 0.011 |
| 3 | mir-424 | 0.51 | 0.012 |
| 4 | mir-452 | 0.523 | 0.016 |
| 5 | mir-92a-1 | 0.53 | 0.02 |
| 6 | mir-365a | 0.532 | 0.022 |
| 7 | mir-491 | 0.57 | 0.026 |
| 8 | mir-552 | 0.58 | 0.032 |
| 9 | mir-1287 | 1.689 | 0.037 |
| 10 | mir-542 | 0.569 | 0.04 |
| 11 | mir-136 | 1.754 | 0.046 |
