# Supplementary material for: MicroRNA-1224 Inhibits Tumor Metastasis in Intestinal-Type Gastric Cancer by Directly Targeting FAK
Source: Front Oncol. 2019 Apr 4;9:222. doi: 10.3389/fonc.2019.00222 (PMC6458237; doi:10.3389/fonc.2019.00222)
Supplement: Table S2 — The correlations of miR-1224 with clinicopathological features of intestinal-type GC. [file Table_2.doc]

Table S2.The correlations of miR-1224 with clinicopathological features of intestinal-type GC

| Variable | Cases | miR-1224 expression(%) | | P value |
| --- | --- | --- | --- | --- |
|  |  | Low | High |  |
| Gender  male  female | 69  21 | 24(72.7%)  9(27.3%) | 45(78.9%)  12(21.1%) | 0.501 |
| Age  <60  ≥60 | 33  57 | 11(33.3%)  22(66.7%) | 22(38.6%)  35(61.4%) | 0.300 |
| T stage  T1/T2/T3  T4a/T4b | 30  60 | 7(21.2%)  26(78.8%) | 23(40.4%)  34(59.6%) | 0.063 |
| Lymph-node metastasis  Absence  Presence | 27  63 | 5(15.2%)  28(84.8%) | 22(38.6%)  35(61.4%) | 0.019 |
| Borrmann category  I/II  III/IV | 20  70 | 8(24.2%)  25(75.8%) | 12(21.1%)  45(78.9%) | 0.726 |
