# Supplementary material for: MicroRNA-1224 Inhibits Tumor Metastasis in Intestinal-Type Gastric Cancer by Directly Targeting FAK
Source: Front Oncol. 2019 Apr 4;9:222. doi: 10.3389/fonc.2019.00222 (PMC6458237; doi:10.3389/fonc.2019.00222)
Supplement: Table S3 — KEGG pathway results of candidate target genes. [file Table_3.DOCX]

Table S3: KEGG pathway results of candidate target genes

| Gene Name | KEGG_PATHWAY |
| --- | --- |
| HGS | Endocytosis  Phagosome  Glutathione metabolism  Metabolic pathways |
| PTK2 | ErbB signaling pathway  Chemokine signaling pathway  PI3K-Akt signaling pathway  Axon guidance  VEGF signaling pathway  Focal adhesion  Leukocyte transendothelial migration  Regulation of actin cytoskeleton  Bacterial invasion of epithelial cells  Amoebiasis  Pathways in cancer  Transcriptional misregulation in cancer  Proteoglycans in cancer  Small cell lung cancer |
| SEC31A | Protein processing in endoplasmic reticulum |
| PLXNA2 | Axon guidance |
| PSEN1 | Wnt signaling pathway  Notch signaling pathway  Neurotrophin signaling pathway  Alzheimer's disease |
